# The influence of sexual prejudice and gender on trait and state-level empathy

**DOI:** 10.3389/fpsyg.2025.1527124

**Published:** 2025-04-01

**Authors:** Seth B. Winward, Roxane J. Itier

**Affiliations:** Face Processing and Social Cognition Lab, Department of Psychology, University of Waterloo, Waterloo, ON, Canada

**Keywords:** empathy, sexual prejudice, gender, sexual orientation, prejudice

## Abstract

A few studies indicate that trait sexual prejudice is negatively related to trait empathy as measured by the Interpersonal Reactivity Index. Whether this association persists at the state level and is modulated by gender remains unknown. Participants read vignettes describing gay/lesbian or straight male and female characters in emotional scenarios and rated their state empathy for each character. Women reported more empathy than men and gay/lesbian targets elicited less empathy than straight targets. In addition, state empathy positively correlated with trait empathy and both negatively correlated with trait sexual prejudice. Results demonstrate that the negative association between empathy and sexual prejudice persists at the state level. We discuss our findings through the lens of social identity theory and gender roles.

## 1 Introduction

Empathy is an essential component of social cognition that is typically divided into affective and cognitive empathy (Decety and Lamm, 2006; Watt, 2007; Batson, 2009; Decety, 2011; Cuff et al., 2016). Affective empathy (also called affective sharing) is defined by spontaneously modulating one’s own emotional state to match the emotions of another person (Decety and Jackson, 2006; Decety and Lamm, 2006; Batson, 2009; Hatfield et al., 2009; van Baaren et al., 2009; Decety, 2011; Cuff et al., 2016). Empathic concern is a motivational construct defined by “feeling for” others, characterized by concerned, caring feelings for others’ wellbeing that prime one toward prosocial behavior to alleviate others’ distress (Davis, 1983; Decety and Lamm, 2006, 2009). Some researchers consider it to be affective (Shamay-Tsoory et al., 2004, 2009; Dziobek et al., 2008; Calabria et al., 2009; Spreng et al., 2009; Harari et al., 2010; Hooker et al., 2010; Bock and Hosser, 2014) while others describe it as cognitive (Decety and Lamm, 2006, 2009; Fabi and Leuthold, 2016; Longobardi et al., 2019), with little consistency across studies on how this variably defined construct fits into the overall taxonomy of empathy. Perspective-taking describes the metacognitive process of consciously thinking about how others are feeling (Davis, 1983; Decety and Jackson, 2006; Decety and Lamm, 2006, 2009; Watt, 2007; Cuff et al., 2016). The present study focused on empathic concern and its association with sexual prejudice. For the purposes of this study, we consider empathic concern to be a cognitive empathy construct, as we used state-level self-report measures that require a degree of cognitive evaluation to complete. We note that this perspective is drawn from the social neuroscience literature on empathy, which is more congruent with the experimental paradigm used in the present study. For an in-depth discussion of empathic concern’s place in this literature, see Decety and Lamm (2009).

Empathy is partially a trait-level construct that is subject to individual differences (Davis, 1983; Baron-Cohen and Wheelwright, 2004; Jolliffe and Farrington, 2006; Spreng et al., 2009; Banissy et al., 2012; Cuff et al., 2016; Vachon and Lynam, 2016). Studies using trait measures such as the Interpersonal Reactivity Index (IRI; Davis, 1983) suggest the ability to empathize is stable across time, with reasonable test–retest reliability, and that women display more empathy than men (Toussaint and Webb, 2005; Sonnby-Borgström et al., 2008; Derntl et al., 2010; Kobach and Weaver, 2012; Groen et al., 2013; Longobardi et al., 2019; Di Tella et al., 2020; Stępień-Nycz et al., 2021). However, many state-level factors also influence empathic responding, including similarity between the empathizer and the person they empathize with (hereafter the target), cognitive load, and moral judgment of the target (Contreras-Huerta et al., 2014; Meiring et al., 2014; Sessa et al., 2014; Cuff et al., 2016; Fabi and Leuthold, 2016; Molenberghs et al., 2016; Fourie et al., 2017; Han, 2018; Suleiman et al., 2018), although the strength of these modulations is not fully understood. Another factor that has received much interest in recent empathy literature is prejudice, with most work focusing on how empathic concern differs based on the race of the target. In brief, individuals who score highly on the IRI and its empathic concern subscale tend to have low scores on trait measures of prejudice and vice versa (Bäckström and Björklund, 2007; Pettigrew and Tropp, 2008; Sidanius et al., 2013; Vanman, 2016; Hudson et al., 2019; Stathi et al., 2021). In research on empathy for physical pain, participants typically underestimate the pain of other-race targets and report less concern for their wellbeing relative to same-race targets (Forgiarini et al., 2011; Kaseweter et al., 2012; Trawalter and Hoffman, 2015; Suleiman et al., 2018). This research clearly indicates a negative association between racial prejudice and empathy at the trait and state level.

In contrast, the association between empathy and sexual prejudice has received little attention. Sexual prejudice is defined as prejudice against others based on their sexual orientation or behavior (Herek, 2000; Herek and McLemore, 2013). Research suggests that sexual prejudice is rooted in the feeling of disgust (Shields and Harriman, 1984; Herek, 2000; Mahaffey et al., 2005; Olatunji, 2008; Inbar et al., 2009; Terrizzi et al., 2010; Herek and McLemore, 2013; Crawford et al., 2014; Kiebel et al., 2017; O’Handley et al., 2017), a negative emotion that could influence empathy. Only a few studies have investigated the association between sexual prejudice and empathy. Castiglione et al. (2013) found a negative correlation between trait empathy and trait sexual prejudice, using measures of affective and cognitive empathy. Poteat et al. (2013) showed that empathic concern was a negative predictor of anti-LGBTQ+ bullying in adolescents. Burke et al. (2015) found that both the empathic concern and the perspective-taking subscales of the IRI were negative predictors of explicit bias against gays and lesbians in medical students. Similarly, in a large sample of college students, Marsden and Barnett (2020) found that lower scores on the IRI empathic concern and perspective-taking subscales predicted higher levels of sexual prejudice.

The studies on sexual prejudice exclusively used trait measures of empathy, with no measures of state empathy. We differentiate between the two not only based on their temporal aspects, but on the ways in which they are measured and the psychological processes involved. Trait empathy scales ask participants to report how often they feel, think, or act in ways that are considered empathic (see Davis, 1983; Spreng et al., 2009). Therefore, trait empathy measures necessarily promote self-reflection about one’s own empathic tendencies, recruiting explicit memory, metacognition, and other processes associated with self-evaluation. State empathy measures, even self-report rating scales, do not encourage this degree of self-reflection and therefore do not recruit additional processes to the same extent. Because of this, state empathy measures are seen by some as a purer measure of empathy that are less confounded by self-evaluative processes, as well as the biases that come with reporting on one’s own emotional and cognitive abilities (Murphy and Lilienfeld, 2019).

It is important to investigate the association between empathy and sexual prejudice at the state level because state-level empathy is closer to what people might feel during everyday experiences. When asked about their empathic tendencies on questionnaires, people’s answers do not always correspond well with how they respond in empathy-inducing scenarios (Murphy and Lilienfeld, 2019; Lima and Osório, 2021). Likewise, when people rate their feelings toward LGBTQ+ people, their responses do not always reflect how they feel and behave in everyday life (Herek and McLemore, 2013; Mahaffey et al., 2005; Morrison et al., 2009; Morrison and Morrison, 2003; Norris, 1992; Ratcliff et al., 2006; Rattazzi and Volpato, 2003). Additionally, trait measures of sexual prejudice lack the implicit nature of many real-world interactions, in which individuals must rely on contextual clues to discern the sexual orientation of others. Therefore, we should not assume that previous trait-level findings easily generalize to state-level contexts.

Although existing research describes empathy as a protective factor against prejudice (Poteat et al., 2013; Burke et al., 2015; Marsden and Barnett, 2020), none of these correlational studies tested the directionality of the association between empathy and sexual prejudice. Even if we assumed that empathy is indeed a protective factor against prejudice at the trait level, this framing is difficult to translate into the state context where the experimenter would have to explicitly ask participants how much sexual prejudice they feel toward a particular target on each trial. Such an approach would produce strong desirability bias, as even very prejudiced individuals typically understand it is not considered acceptable to say one is prejudiced against a particular minority group (Herek and McLemore, 2013; Morrison and Morrison, 2003; Norris, 1992; Ratcliff et al., 2006; Rattazzi and Volpato, 2003).

Existing state-level research on empathy and racial prejudice typically uses measures of empathy as the dependent variable and manipulates characteristics of targets to make them representative of a specific minority group (Forgiarini et al., 2011; Kaseweter et al., 2012; Trawalter and Hoffman, 2015; Suleiman et al., 2018). Such an approach frames prejudice as interacting with others’ individual differences to dampen empathic responding. Although this view contrasts with the limited research on empathy and sexual prejudice, it is consistent with the broader trait prejudice literature (Sidanius et al., 2013; Vanman, 2016; Hudson et al., 2019). We adopted this theoretical framing for the present study because it is more congruent with state-level experimental paradigms. However, we did not explicitly test this causal assumption itself with this experiment; further research will be needed to empirically assess the causality of the association between empathy and sexual prejudice.

In existing work, trait empathy is most often measured by the IRI, despite recent psychometric evaluations showing issues with the IRI factor structure such that two of its subscales (Personal Distress and Fantasy) do not appear to map onto empathy-specific constructs, unlike its Empathic Concern and Perspective-Taking subscales (Baron-Cohen and Wheelwright, 2004; Spreng et al., 2009; Vachon and Lynam, 2016; Murphy and Lilienfeld, 2019; Lima and Osório, 2021). The IRI also has relatively low convergent validity with other measures of empathy (Murphy and Lilienfeld, 2019; Lima and Osório, 2021). The Toronto Empathy Questionnaire (TEQ) is a unidimensional scale of trait empathy most closely associated with empathic concern and shows greater convergent validity with state empathy than the IRI (Spreng et al., 2009; Lima and Osório, 2021). However, no study has used the TEQ in sexual prejudice research.

Likewise, no empathy study has investigated the gender effects associated with sexual prejudice. Sexual prejudice for same-gender targets is greater than for other-gender targets in heterosexual individuals (Kite and Whitley, 1996; Herek, 2000, 2002; Mahaffey et al., 2005; Herek and Gonzalez-Rivera, 2006; Ahrold and Meston, 2010; Herek and McLemore, 2013; Monto and Supinski, 2014), especially among male participants (Herek, 2000; Parrott and Gallagher, 2008; Herek and McLemore, 2013). For instance, a heterosexual male participant will likely express more prejudice and disgust toward a gay man than toward a lesbian, whereas the converse is true with heterosexual female participants. Despite this well documented gender bias, no study has yet investigated whether the effect of sexual prejudice on empathy depends on the gender of the participant and of their target at either the trait or state level.

The present study investigated whether both trait empathy, as measured by the TEQ, and state empathy, are negatively associated with sexual prejudice and whether these associations are modulated by target and participant gender. We designed empathy-inducing vignettes displaying negative or neutral scenarios and asked participants to rate their affect and level of empathic concern (state empathy) for the target character. We manipulated the sexual orientation and gender of the target character in each vignette. Since the TEQ’s stability over time is still poorly understood, we administered it twice to evaluate its test–retest reliability. We conceptualized the state-level process as follows: contextual clues (e.g., name, gendered pronouns, gendered pronouns of the target’s partner) indicate whether the target is straight or gay/lesbian; the latter would activate the participant’s latent sexual prejudice, especially so if the target is the same gender as the participant. The degree to which state-level sexual prejudice is activated would determine the degree to which the state-level empathic response to the target is dampened. Furthermore, state empathy should be dampened more if the participant is a man, compared to a woman, as men typically exhibit greater sexual prejudice than women.

Based on previous literature, we predicted that TEQ scores, our trait empathy measure, would be positively associated to empathy ratings of the vignette, our state empathy measure (Spreng et al., 2009; Lima and Osório, 2021). We expected both state and trait empathy scores to be higher for women, and for TEQ scores to be stable across time. In line with existing research (Poteat et al., 2013; Burke et al., 2015; Marsden and Barnett, 2020), trait sexual prejudice as measured by the Modern Homonegativity Scale (MHS; Morrison and Morrison, 2003) was expected to be negatively related to trait empathy. We hypothesized that state-level empathy has a similar association to sexual prejudice, but that, in addition, participants would be even less empathetic for gay/lesbian characters relative to heterosexual characters. We also hypothesized that this association would be stronger when participants and targets were of the same gender, based on the gender effects observed in the literature on sexual prejudice (Kite and Whitley, 1996; Herek, 2000, 2002; Mahaffey et al., 2005; Herek and Gonzalez-Rivera, 2006; Ahrold and Meston, 2010; Herek and McLemore, 2013; Monto and Supinski, 2014).

## 2 Method

### 2.1 Participants

We recruited a University of Waterloo (UW) undergraduate student group (*n* = 281) through the SONA system in exchange for course credit, and an Amazon’s Mechanical Turk (hereafter MTurk) group (*n* = 225) through the CloudResearch platform in exchange of monetary compensation ($CAN3.00 for completing the prerequisite study and $CAN12.00 for completing the main study, detailed below). Participants were required to be 18–30 years of age, be living in the United States or Canada, and be fluent English speakers. All participants provided informed written consent prior to participation. The study was conducted according to the Declaration of Helsinki and was approved by the UW Research Ethics Board. After exclusion (see Results), 87 SONA participants and 114 MTurk participants remained, combined into a single group (*N* = 201; 18 to 47 years old, *M* = 24.08, *SD* = 4.46; Table 1). Our final sample size greatly exceeded typical sample sizes in state empathy studies using neuroimaging (Range = 12–48, *M* = 30.57, *SD* = 12.57) (Contreras-Huerta et al., 2014; Meiring et al., 2014; Sessa et al., 2014; Fabi and Leuthold, 2016; Molenberghs et al., 2016; Fourie et al., 2017; Suleiman et al., 2018) and was near the average for studies using empathy-inducing vignettes (Range = 68–350, *M* = 213.00, *SD* = 108.61) (Arditte Hall et al., 2018; Hein et al., 2018; Vera Cruz and Mullet, 2019; McGrath and Haslam, 2020; Gehenne et al., 2021).

**Table 1:** Demographic information for the final combined sample.

Gender	*n*_SONA_	*n*_MTurk_	*N*_Total_	Ethnicity	*n*_SONA_	*n*_MTurk_	*N*_Total_
Male	46	60	106	Black/African	2	7	9
Female	41	54	95	East Asian	23	9	32
				Hispanic	2	8	10
				Middle Eastern	6	1	7
				Mixed	2	4	6
				South Asian	18	4	22
				Southeast Asian	3	6	9
				White	30	75	105
				Declined to answer	1	0	1

All demographic information was chosen from lists of possible options. *N* = 201.

### 2.2 Materials

#### 2.2.1 Mass testing/prerequisite study

**Figure 1:**
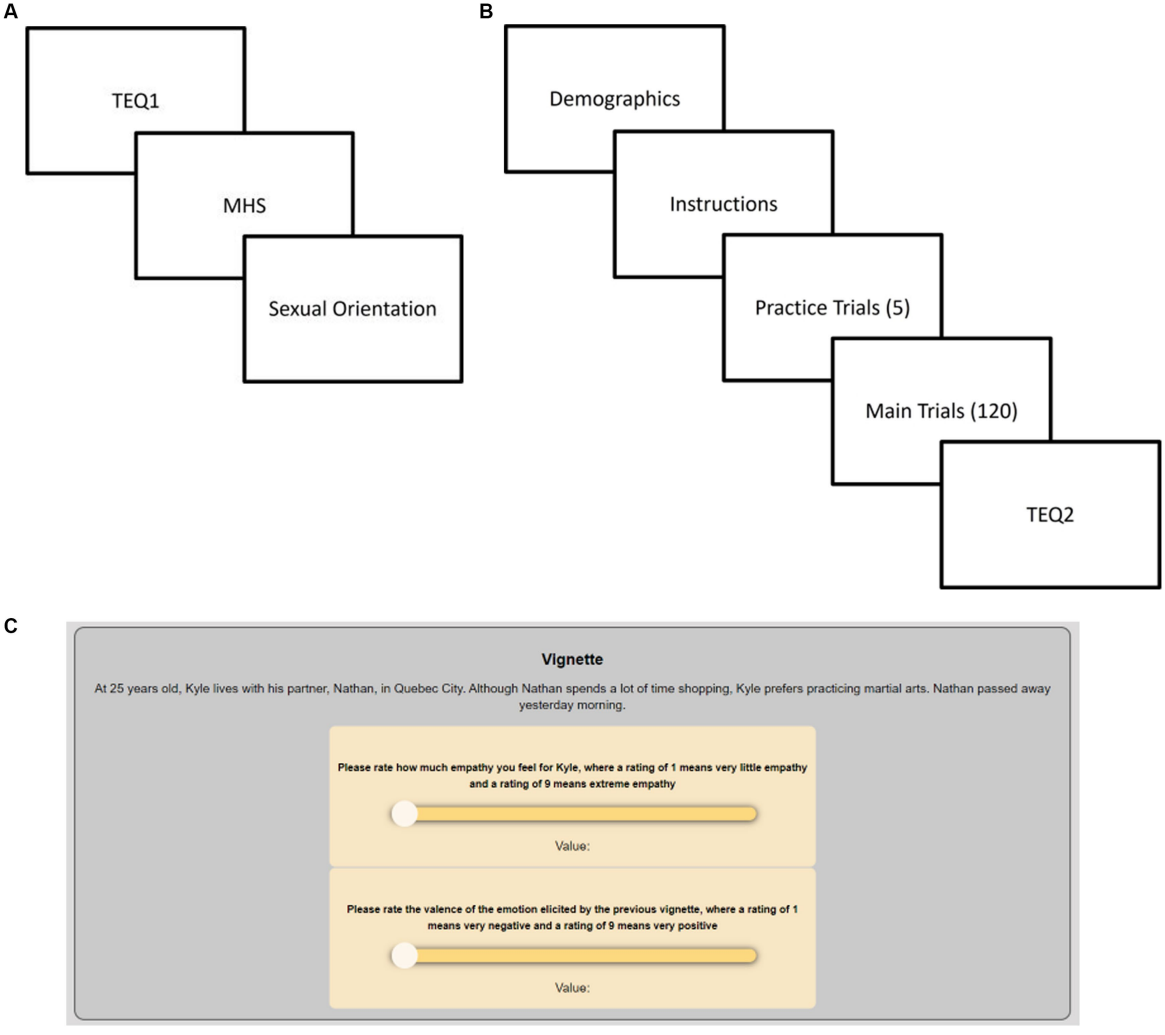
**(A)** Materials and procedure for the Mass Test/Prerequisite Study phase. **(B)** Materials and procedure for the main study phase, administered a few weeks later. **(C)** Sample trial in the Negative Male Gay/Lesbian condition; participants moved sliders to a discrete value between 1 and 9 before moving to the next screen with the memory-related questions.

All participants completed a preliminary series of scales prior to the main study. SONA participants completed these scales as part of the Mass Testing process at UW at the beginning of every semester. MTurk participants completed a brief prerequisite study, created to mimic the Mass Testing process. All questionnaires were presented in semi-random (SONA) or random (MTurk) order using the Qualtrics survey platform (Figure 1A). Only the TEQ, MHS, and sexual orientation questionnaires were analyzed (see Table 2 for score range).

**Table 2:** Descriptive information for TEQ and MHS scores.

Scale	Minimum	Maximum	Mean	*SD*	Cronbach’s *α*
TEQ1	0.93	4.00	2.84	0.61	0.88
MHS	1.00	4.75	2.47	0.88	0.90
TEQ2	1.20	3.47	2.31	0.48	0.58

Data is derived from the final combined sample. *N* = 201.

##### 2.2.1.1 Toronto empathy quotient (TEQ)

The TEQ (Spreng et al., 2009) is a measure of trait empathy consisting of 16 statements such as “When someone else is feeling excited, I tend to get excited too” and “It upsets me to see someone being treated disrespectfully”; participants are asked to rate how frequently they feel or act in the manner described by the statement. Possible responses range from 0 (Never) to 4 (Always). Each participant’s responses were averaged across all items to mitigate missing items effects; scores ranged from 0 to 4, with high scores indicating high trait empathy. Psychometric studies suggest that the TEQ is a sound measure of empathy, with good construct validity and internal consistency (Totan et al., 2012; Kourmousi et al., 2017; Xu et al., 2020). Note that due to a clerical error, the TEQ was presented to participants without the final item, thus scores were computed based on 15 items^1^.

##### 2.2.1.2 Modern homonegativity scale (MHS)

The MHS (Morrison and Morrison, 2003) is a measure of trait sexual prejudice consisting of 12 statements such as “many homosexuals use their sexual orientation so that they can obtain special privileges” and “Homosexuals seem to focus on the way in which they differ from heterosexuals, and ignore the ways in which they are the same.” Participants are asked to rate their level of agreement with these statements on a scale from 1 (Strongly Disagree) to 5 (Strongly Agree). Participants’ responses were averaged, resulting in possible MHS scores between 1 and 5, with high scores indicating high levels of sexual prejudice. The MHS has good construct validity and reliability, having been validated several times since its original publication (Grey et al., 2013; Morrison et al., 2009; Morrison et al., 2005; Peterson et al., 2017; Romero et al., 2015; Rye and Meaney, 2010).

##### 2.2.1.3 Sexual orientation questionnaire

A brief questionnaire was presented alongside the Mass Testing/Prerequisite Study scales, asking participants to identify their sexual orientation and gender identity.

#### 2.2.2 Main study

Participants were invited to take part into a study on memory for social information. The order of materials in the main study phase is depicted in Figure 1B. The study was taken within 12 weeks of Mass Testing for SONA participants and within 1–2 weeks of the prerequisite study for MTurk participants.

##### 2.2.2.1 Demographics questionnaire

All participants completed a simple demographics questionnaire on gender, age, and ethnicity which was compared to the information collected during Mass Testing/Prerequisite Study.

##### 2.2.2.2 Vignettes

The main study included 125 brief written vignettes based on the empathy-inducing stimuli developed by McCrackin and Itier (2021a, 2021b). Five of these were practice trials, with the target gender, partner gender, and valence selected at random. The positive condition consisted of 24 trials [6 × 2 gender (male/female) × 2 sexual orientation (gay/lesbian, heterosexual)] and was included to prevent the study from feeling overwhelmingly negative. Positive trials were not analyzed. The remaining 96 vignettes were divided evenly into eight conditions according to a 2 valence (neutral, negative) × 2 target gender (male, female) × 2 target sexual orientation (gay/lesbian, heterosexual) design. The 12 vignettes making up each of the eight conditions were selected at random from 25 possible generic vignettes. For instance, one generic vignette was “(Character name) is a (age) year old living in (location) with [his/her] partner, (partner name). Outside of work, (character name) enjoys (hobby) and taking care of [his/her] pets. (Character name)’s pet dog was {fed/killed} yesterday afternoon.”

In each vignette, the character’s and partner’s names were randomly selected from a list of masculine or feminine names which implied the sexual orientation of the target. The vignette valence was determined by the final sentence, with a key word or phrase changing to make the scenario negative or neutral. The target’s age, location, and hobby were selected at random from lists of 30 options each, to create variety (see Figure 1C for an example). The vignettes making up each condition were sorted at random such that there were always 12 different vignettes from each condition for every participant, all intermixed at random along with the positive trials.

##### 2.2.2.3 Vignette questions

Below each vignette, participants were asked to rate how much empathy they felt for the character, and the valence of the emotion elicited by the vignette, on scales from 1/very little empathy/very negative to 9/extreme empathy/very positive. These rating scales were identical to those used by McCrackin and Itier (2021a, 2021b). Participants dragged the sliding scale to their response number using the mouse. Next, they were presented with a second screen asking them to recall two of the three following details: the target’s age, the target’s location, or the target’s hobby. Participants typed in their answers in the two text boxes provided. These questions ensured participants were paying attention to the vignettes and gave credence to the deception of studying memory for social information. Responses to these questions were used for data screening.

### 2.3 Procedure

Within 12 weeks of the beginning of each term and prior to the main study, SONA participants completed the Mass Testing survey that included the demographics questionnaire, TEQ (hereafter TEQ1), MHS, sexual orientation questionnaire, and many other (not analyzed) questionnaires. The study spanned the winter 2021, spring 2021, fall 2021, and winter 2022 terms, i.e., during the COVID-19 pandemic in Ontario.

During the same time, MTurk Participants were recruited to the Prerequisite Study through an online notice on the MTurk platform, which informed them that completing this study would make them eligible for the better remunerated main study. A link directed them to the Prerequisite Study hosted on Qualtrics. Participants were first presented with an information and consent form, followed by the demographics questionnaire, TEQ1, MHS, sexual orientation questionnaire, and 5 other (not analyzed) questionnaires, all presented in random order. One week later, they received an email mentioning the main study was open. A final email invitation was sent another week later to those who had not yet taken the study. The maximum latency period between the Prerequisite Study and the main experiment was therefore shorter for the MTurk sample than the SONA sample.

The main study was hosted on Qualtrics and was identical for SONA and MTurk participants. Instructions on how to respond to the vignettes and their questions were provided, followed by five practice trials, then 120 experimental trials in random order (the 96 main vignettes mixed with the 24 positive vignettes). Empathy and valence ratings, as well as the memory questions, were recorded after each vignette. Every 25 vignettes, they were asked to solve a simple algebra problem as an attention check. At the same time, they received a reminder to take a brief break as needed to prevent fatigue. After the main task, participants were asked to complete the TEQ (hereafter TEQ2). The procedure is summarized in Figure 1.

### 2.4 Data screening and preprocessing

Participants who did not consent to having their data used after debriefing (*n* = 8) and those who did not complete the study (*n* = 141) were rejected. All non-heterosexual participants were removed (*n* = 47).

We calculated the longest sequence of identical responses for each participant and defined a cutoff point as the third quartile plus the interquartile range of this variable (16 for SONA, 12 for MTurk). Those who repeated the same response to the empathy rating question for more than that number of trials in a row were rejected (*n* = 41). We also calculated the number of trials with incorrect responses for each participant and defined a cutoff point as the third quartile plus the interquartile range of this variable (14 trials for SONA, 6 trials for MTurk). We rejected those who failed to answer at least one memory question correctly on that calculated number of trials or more (*n* = 29), as such participants were consistently not paying attention to the vignettes. We rejected participants who took longer than the median time plus half the median time to complete the study (*n* = 8) as we did not deem it reasonable for a single session to take longer than this (1 h 50 min for SONA, 1 h 25 min for MTurk). Similarly, any participant who completed the study in less than half the median time (36 min and 42 s for SONA, 28 min and 33 s for MTurk) was rejected (*n* = 36) as they were likely not reading all the vignettes and question prompts.

For the remaining participants, trials without at least one correctly answered memory question were removed. We reasoned that if participants could not recall the answer for either question, they were not paying attention during that trial. The number of trials rejected per participant ranged from 0 to 13 in the SONA sample (*M* = 4.48, *SD* = 3.28) and from 0 to 6 (*M* = 2.61, *SD* = 1.54) in the MTurk sample. Thus, our final sample consisted of 87 SONA participants and 114 MTurk participants, for a total of 201 participants. Given their similarity (see Data Analysis section below), the two samples were combined. Data from this study are available upon request from the corresponding author.

For all dependent variables, univariate outliers (>3 standard deviations above or below the mean of a particular variable) were winsorized (26 total) using scores corresponding to 3 SDs above or below the mean. Descriptive statistics for all variables of interest revealed no skew values greater than ±3 and no kurtosis values greater than ±10, indicating approximately normal distributions for all variables (Kline, 1998). Visual inspection of Q-Q plots for each variable was consistent with this assessment; the assumption of normality was not violated for any of the present analyses. Since each factor in the ANOVA had no more than two levels, the sphericity of the data was not a cause for concern. Tests of homogeneity of variance were conducted as part of the primary ANOVA analysis and are described below. All results were calculated using SPSS version 28.0.1.0 with *α* = 0.05; Bayes factors (denoted *BF*) were obtained for each correlation using JASP version 0.16.3 and interpreted as described by Lee and Wagenmakers (2013).^2^ Observed power was calculated with G*Power 3.1.9.7 (Faul et al., 2007).

### 2.5 Data analysis and specific predictions

#### 2.5.1 Analyses of mean empathy ratings (state empathy)

To ensure that group status did not influence our results, we first ran an omnibus 2 × 2 × (2 × 2 × 2) mixed model ANOVA with vignette valence (negative, neutral), target gender (male, female) and target sexual orientation (gay/lesbian, heterosexual) as within-subject factors and group (SONA, MTurk) and participant gender (men, women) as between-subject factors. There was no main effect of group, *F*(1, 197) = 2.72, *MSE* = 20.80, *p* = 0.101, *η*^2^ = 0.01, and no key interaction with group^3^, indicating that empathy ratings did not vary meaningfully between SONA or MTurk samples. We thus re-ran the omnibus ANOVA without the group factor to increase power and report these results below. We predicted:

*P1*: There would be a main effect of valence such that empathy ratings would be higher for negative than neutral vignettes (manipulation check).

*P2*: We predicted a vignette valence by participant gender interaction such that women would display more empathy in the negative condition than men.

*P3*: We predicted a significant interaction between vignette valence and target sexual orientation, such that participants would express less empathy for gay/lesbian targets relative to heterosexual targets within negative vignettes.

*P4*: Following the sexual prejudice literature, this decreased empathy for gay/lesbian targets would be more pronounced for same-gender compared to other-gender gay/lesbian targets, such that a four-way interaction was expected.

#### 2.5.2 Associations between empathy and sexual prejudice

Pearson’s correlations were calculated to determine the associations between participants’ mean state empathy ratings, average scores on the MHS, and TEQ1. For each participant, we computed the mean state empathy score across (i) all 8 negative conditions, (ii) the 4 negative conditions with gay/lesbian targets and (iii) the 4 negative conditions with heterosexual targets. According to Kline’s (1998) normality criteria, all aggregate scores were normally distributed. We predicted:

*P5*: Trait sexual prejudice would be negatively correlated with participants’ empathy ratings and this association would be stronger for gay/lesbian characters than for heterosexual characters.

*P6*: TEQ scores would be positively correlated with average empathy ratings across all negative conditions.

*P7*: TEQ scores would be negatively correlated with MHS scores.

#### 2.5.3 TEQ stability analyses

To evaluate the stability of the TEQ, we first conducted a 2 (Time: TEQ1, TEQ2) × 2 (Participant Gender: male, female) mixed-model ANOVA. We predicted:

*P8*: Trait empathy would be stable over time and thus there would be no effect of time.

*P9*: Women would score higher than men on the TEQ.

*P10*: If TEQ was stable as expected, then we predicted a strong correlation between TEQ1 and TEQ2 scores.

## 3 Results

### 3.1 Analyses of mean empathy ratings (state empathy)

**Figure 2:**
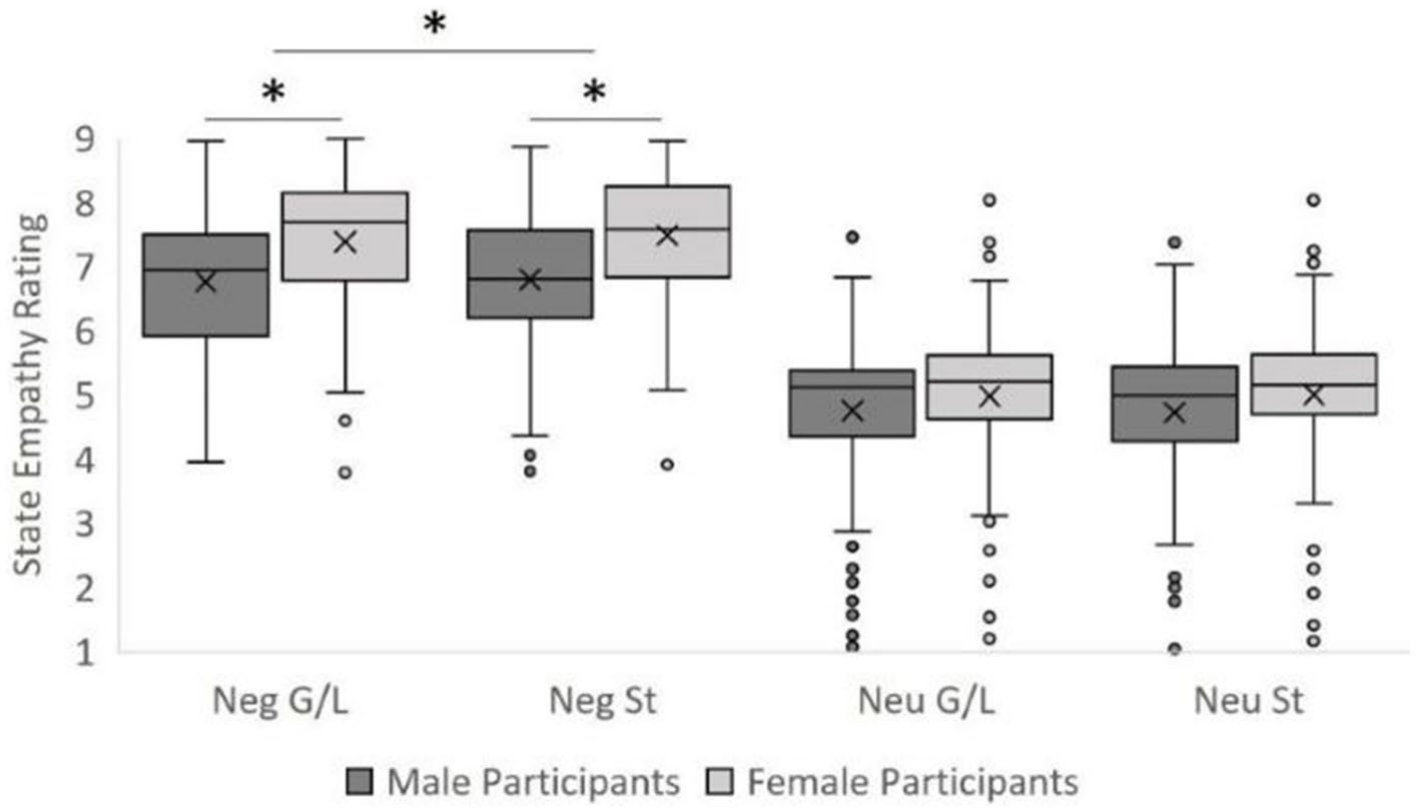
Effects of vignette valence, participant gender, target gender, and target orientation. Neg, negatively valenced vignettes. Neu, neutrally valenced vignettes. G/L, gay/lesbian target characters. St, straight target characters. Significant differences between negative conditions are denoted with an asterisk (*).

Homogeneity of variance was not violated in any condition (all Levene’s tests *p*s > 0.05). The omnibus ANOVA revealed the expected significant effect of vignette valence (prediction P1), *F*(1, 199) = 619.24, *MSE* = 2020.80, *p* < 0.001, *η*^2^ = 0.76, driven by higher empathy scores for negative (*M* = 7.11, *SD* = 0.07) relative to neutral vignettes (*M* = 4.87, *SD* = 0.09), indicating that the manipulation check was successful (*BF* = 1.29 × 10^13^; Power = 1)^4^. Higher empathy scores were found for women (*M* = 6.20, *SD* = 0.10) than men (*M* = 5.77, *SD* = 0.10), *F*(1, 199) = 11.03, *MSE* = 84.76, *p* = 0.001, *η*^2^ = 0.05 (*BF* = 10.66, Power = 0.91). These main effects were qualified by the expected significant interaction between vignette valence and participant gender (P2), *F*(1, 199) = 4.79, *MSE* = 15.62, *p* = 0.03, *η*^2^ = 0.02 (*BF* = 1.83, Power = 0.59; see Figure 2). Simple effects testing revealed higher empathy ratings for women (*M* = 7.44, *SD* = 0.11) than men (*M* = 6.78, *SD* = 0.10) within negative vignettes, *F*(1, 199) = 19.81, *MSE* = 21.65, *p* < 0.001, *η*^2^ = 0.09. By contrast, in neutral vignettes men (*M* = 4.73, *SD* = 0.13) had very similar empathy ratings compared to women (*M* = 5.00, *SD* = 0.10), *F*(1,199) = 2.10, *MSE* = 3.45, *p* = 0.149, *η*^2^ = 0.01. There was also an unexpected, small main effect of target gender such that female targets generally exhibited greater empathy (*M* = 6.02, *SD* = 0.07) relative to male targets (*M* = 5.96, *SD* = 0.07), *F*(1,199) = 3.94, *MSE* = 1.48, *p* = 0.049, *η*^2^ = 0.02 (*BF* = 0.11, Power = 0.51).

The predicted interaction between vignette valence and target sexual orientation (P3) was also significant, *F*(1, 199) = 4.10, *MSE* = 0.58, *p* = 0.044, *η*^2^ = 0.02 (*BF* = 0.12, Power = 0.52). This effect was driven by an effect of target sexual orientation in the negative condition only (*F*(1, 199) = 6.01, *MSE* = 1.07, *p* = 0.015, *η*^2^ = 0.03, Power = 0.68), with slightly more empathy for straight targets (*M* = 7.14, *SD* = 0.07) relative to gay/lesbian targets (*M* = 7.08, *SD* = 0.08) (see Figure 2). The predicted four-way interaction (P4) was not significant, *F*(1, 199) = 0.03, *MSE* = 0.01, *p* = 0.856, *η*^2^ = 0.00 (*BF* = 2.69 × 10^−7^, Power = 0.05, suggesting the test was greatly underpowered). No other significant effects or interactions were observed.

### 3.2 Associations between empathy and trait sexual prejudice

**Figure 3:**
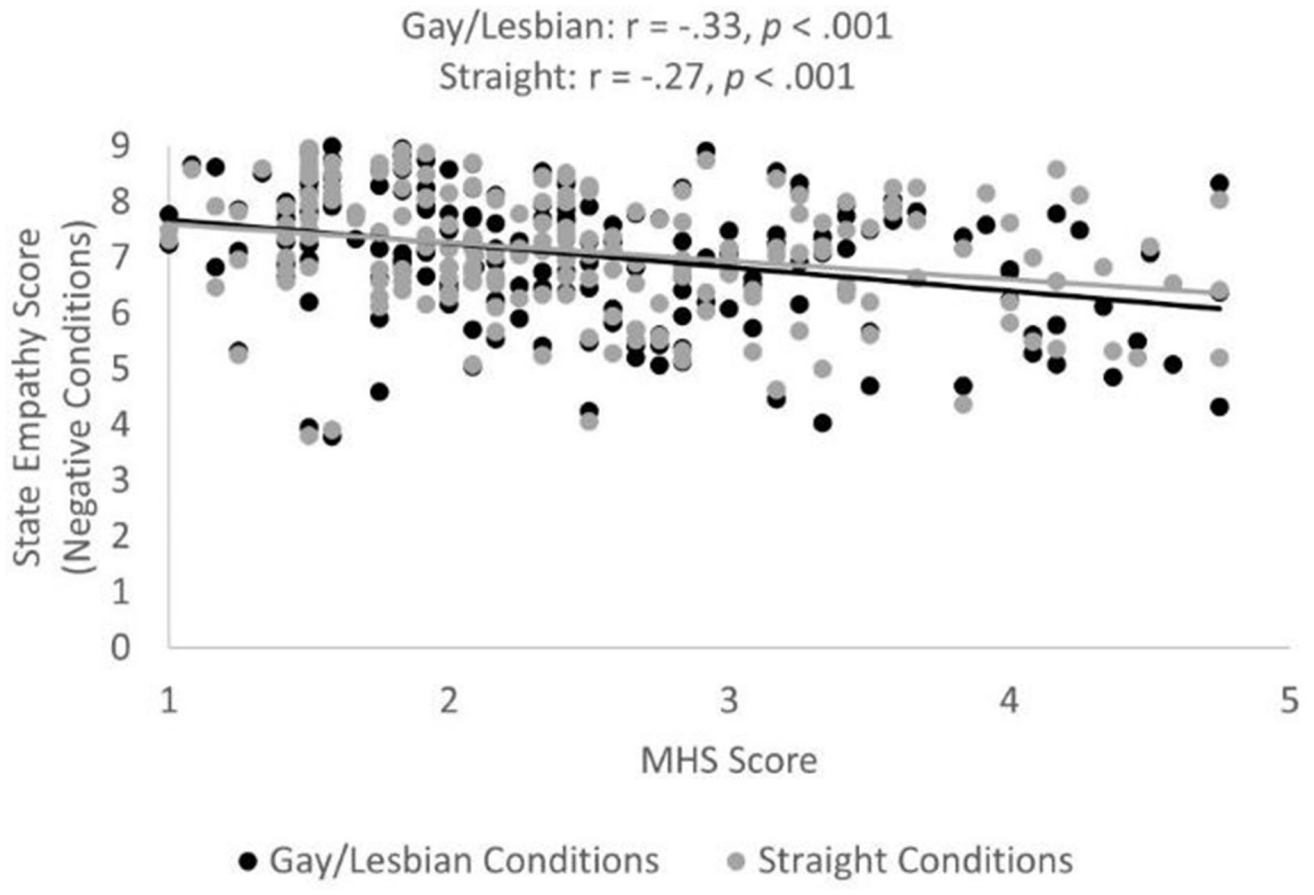
Significant correlations between MHS and mean state empathy scores. Note MHS scores reflect trait sexual prejudice. Mean state empathy scores averaged across gay/lesbian negative conditions are denoted with black dots, state empathy scores averaged across straight negative conditions are denoted with grey dots.

**Figure 4:**
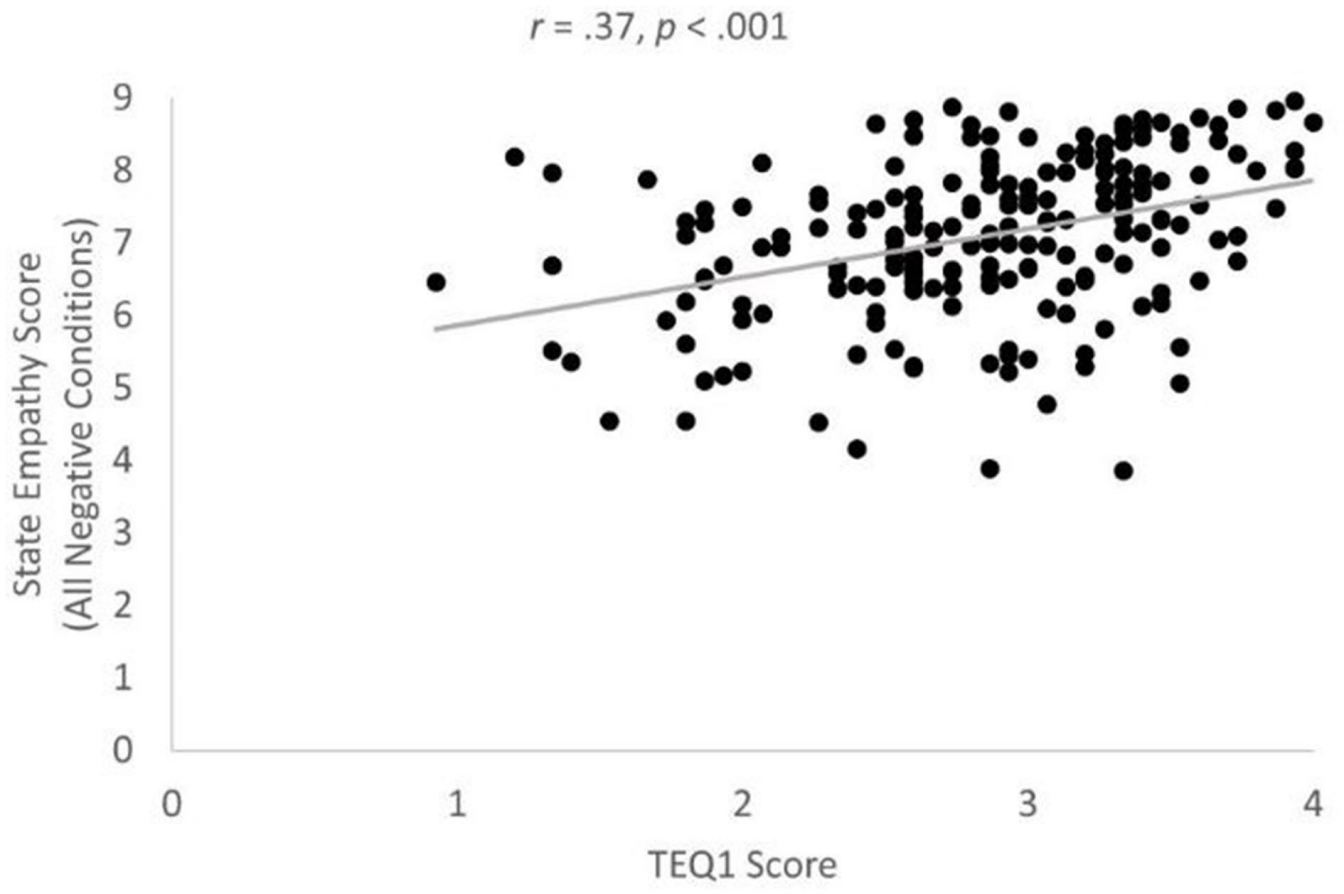
Significant correlation between TEQ1 and mean state empathy scores. Note TEQ1 refers to TEQ scores obtained during Mass Testing/prerequisite study. Mean state empathy scores were averaged across all negative conditions during the experimental study.

**Figure 5:**
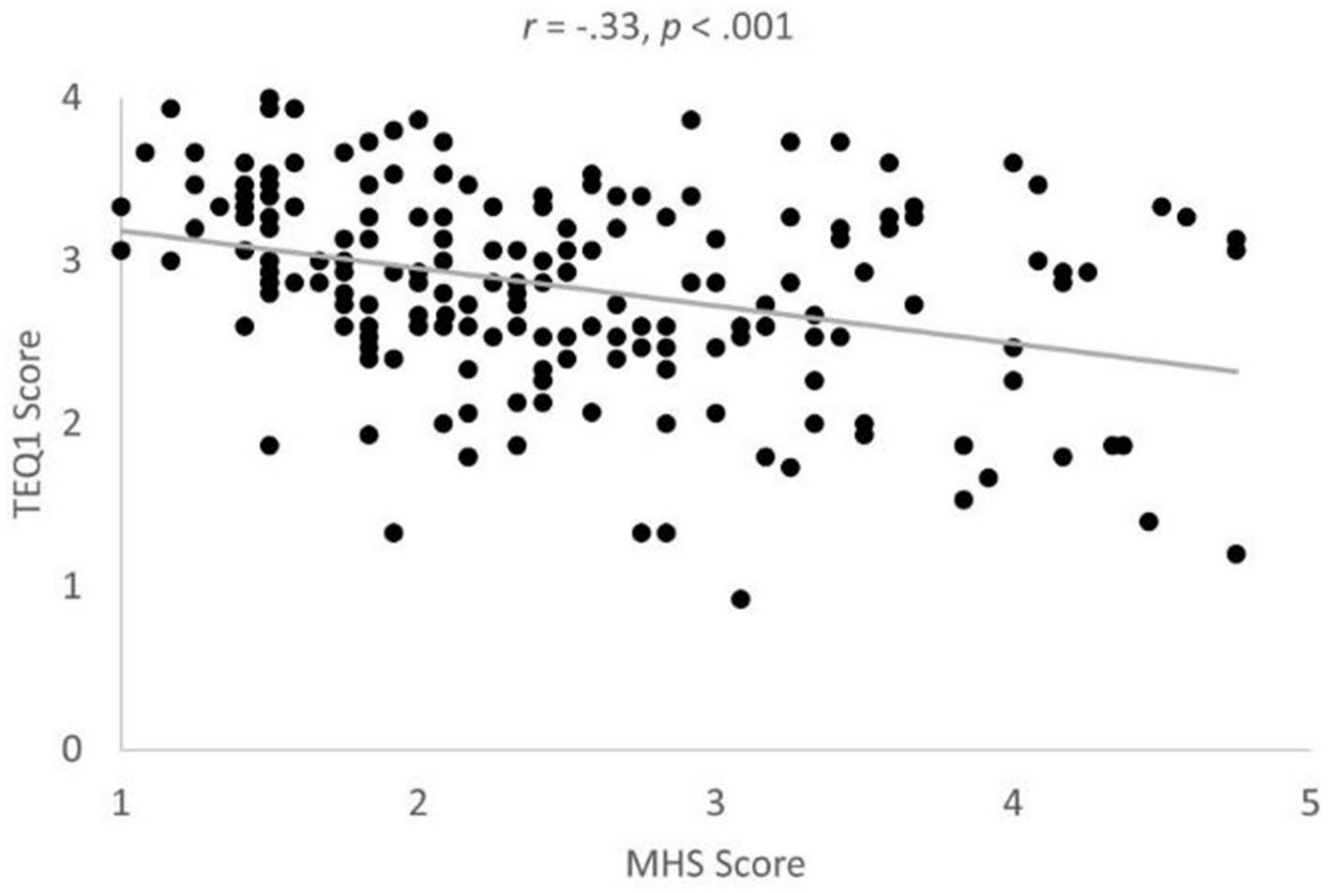
Significant correlation between TEQ1 and MHS scores. Note Scores on both scales were obtained during Mass Testing/prerequisite study. Note TEQ scores were lower in the experiment (TEQ2) than during the Mass Testing/Prerequisite study (TEQ1).

The correlation between MHS scores and state empathy scores was significant in both the average gay/lesbian negative condition (Figure 3), *r* = −0.33, *p* < 0.001, *BF* = 10053.86, Power = 1.00, and in the average heterosexual condition, *r* = −0.27, *p* < 0.001, *BF* = 249.39, Power = 0.99, providing partial support for prediction P5. In line with prediction P6, the correlation between TEQ1 scores and average state empathy scores across all negative conditions was positive and significant, *r* = 0.37, *p* < 0.001 (Figure 4); *BF* = 357154.03, Power = 1.00. The correlation between TEQ1 scores and MHS scores was negative and significant, *r* = −33, *p* < 0.001, in line with prediction P7 (Figure 5); *BF* = 15639.56, Power = 1.00.

### 3.3 TEQ stability analyses

**Figure 6:**
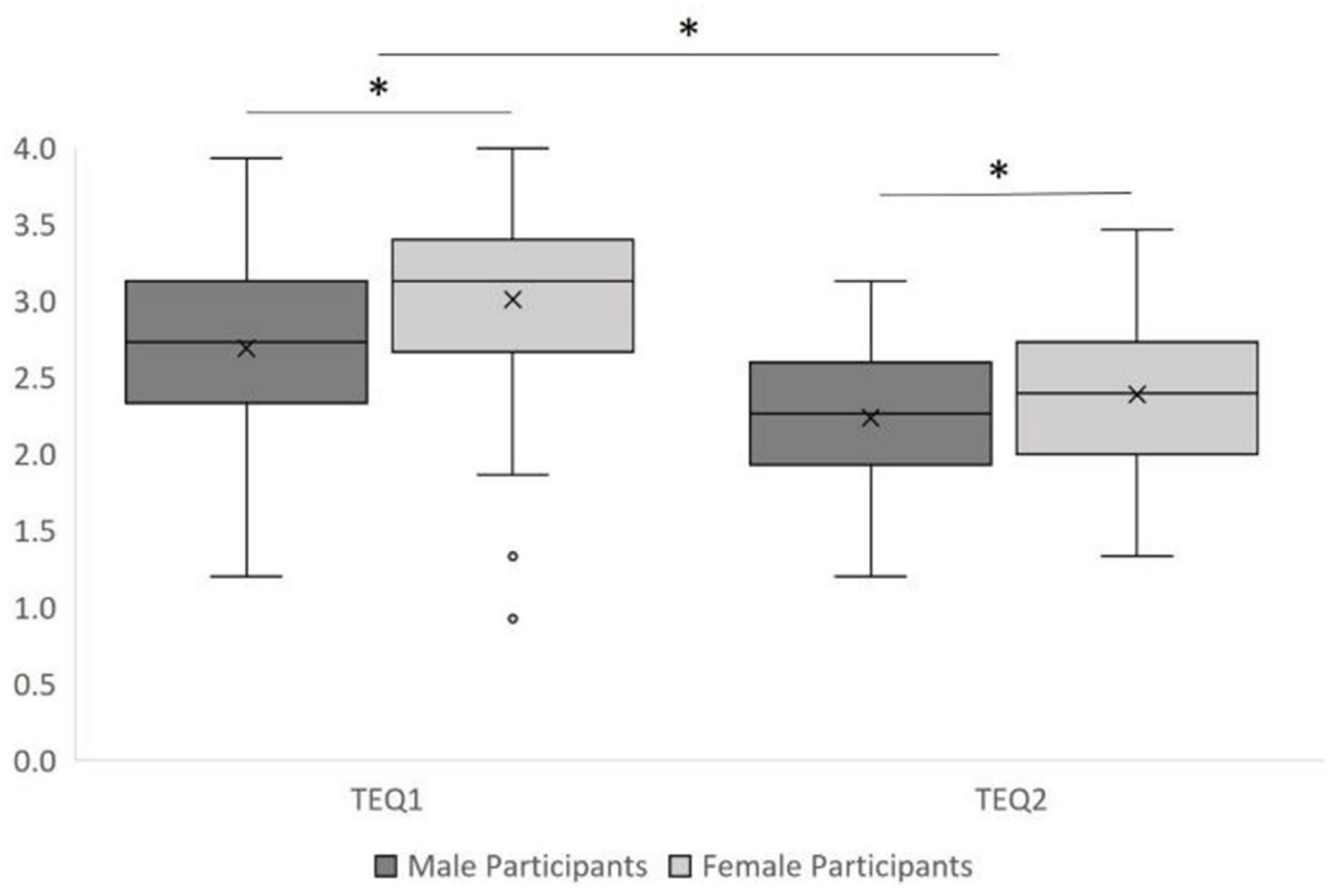
TEQ scores during mass testing/prerequisite study and during main study. Note TEQ scores were lower in the experiment (TEQ2) than during the Mass Testing/Prerequisite study (TEQ1). Significant differences are denoted with an asterisk (*).

**Figure 7:**
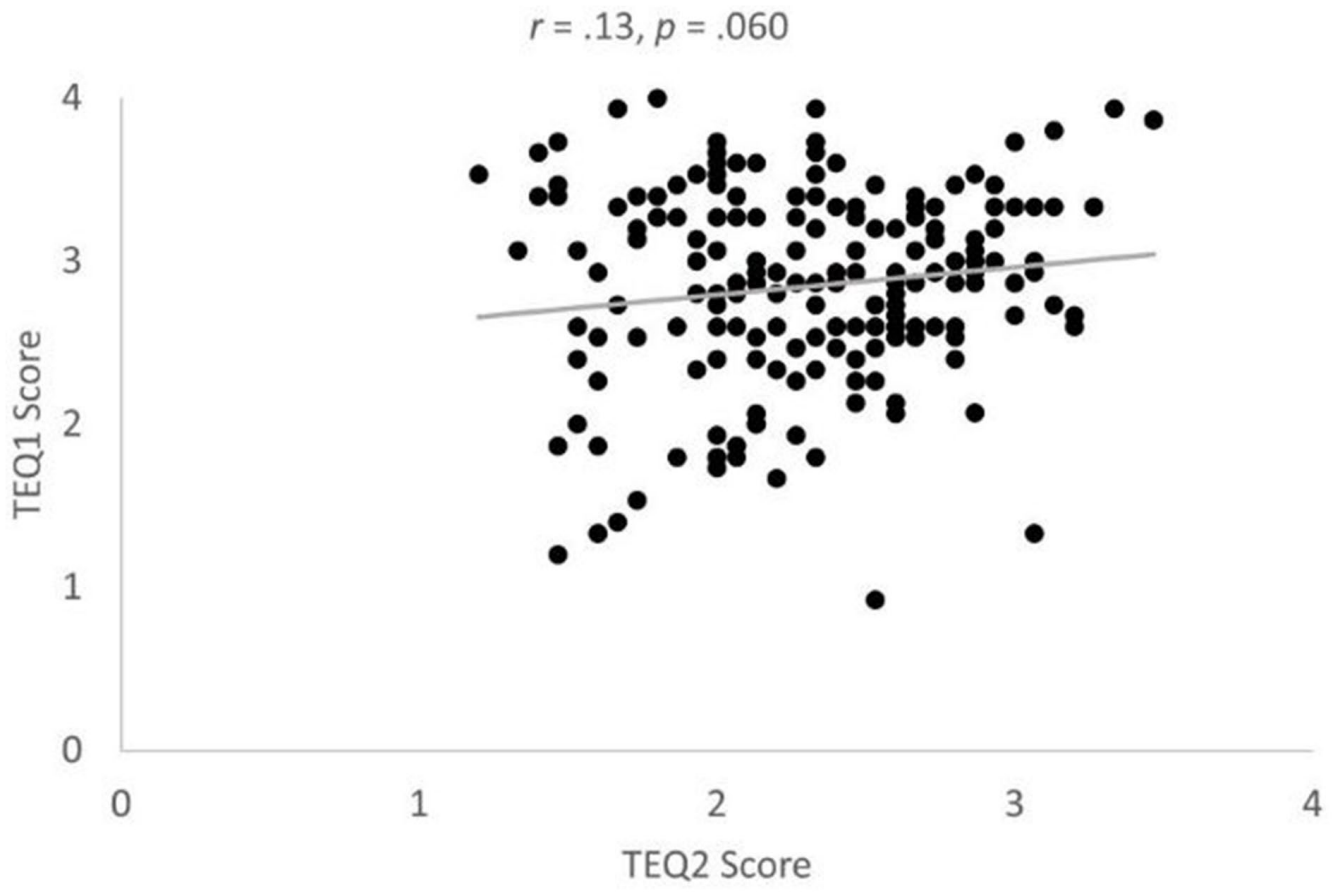
Non-significant correlation between TEQ1 and TEQ2 scores. Note TEQ1 refers to TEQ scores obtained during Mass Testing/Prerequisite Study and TEQ2 refers to TEQ scores taken after the experiment.

A significant main effect of time was found, *F*(1, 198) = 111.661, *MSE* = 29.22, *p* < 0.001, *η*^2^ = 0.36, such that participants’ TEQ scores were higher during Mass Testing/prerequisite study (TEQ1, *M* = 2.85, *SD* = 0.04) compared to after the experiment (TEQ2, *M* = 2.31, *SD* = 0.03) contrary to our expectation (P8) that their scores would not change significantly over time (*BF* = ~∞; Power = 1.00). We also observed the expected significant main effect of participant gender (P9) such that women had greater TEQ scores (*M* = 2.70, *SD* = 0.04) than men (*M* = 2.46, *SD* = 0.04), *F*(1, 198) = 18.05, *MSE* = 5.69, *p* < 0.001, *η*^2^ = 0.08 (*BF* = 366.30; Power = 0.99). The interaction between time and participant gender was not significant, *F*(1, 198) = 2.38, *MSE* = 0.62, *p* = 0.125, *η*^2^ = 0.01 (*BF* = 1.49, Power = 0.34) (see Figure 6) for depiction of results. In contrast to prediction P10, the correlation between the two TEQ scores was not significant, *r* = 0.13, *p* = 0.060 (*BF* = 0.99, Power = 0.60) (Figure 7).

## 4 Discussion

A handful of studies report that high empathy is associated with low sexual prejudice (Castiglione et al., 2013; Poteat et al., 2013; Burke et al., 2015; Marsden and Barnett, 2020). Furthermore, the sexual prejudice literature suggests that individuals are more prejudiced against LGBTQ+ people if they are of the same gender (Kite and Whitley, 1996; Herek, 2000, 2002; Mahaffey et al., 2005; Herek and Gonzalez-Rivera, 2006; Parrott and Gallagher, 2008; Ahrold and Meston, 2010; Herek and McLemore, 2013; Monto and Supinski, 2014). The present study tested whether these under-studied associations between empathy and sexual prejudice persist when measuring state empathy ratings in response to vignettes. We also investigated whether existing trait empathy findings using the Interpersonal Reactivity Index (IRI) would replicate with the Toronto Empathy Quotient (TEQ) which has stronger convergent validity than the IRI (Spreng et al., 2009; Lima and Osório, 2021). We discuss our findings below.

### 4.1 State empathy and its association with sexual prejudice

Heterosexual participants’ empathy ratings were significantly lower for negative vignettes with gay/lesbian target characters relative to straight targets. This confirmed our predictions (P3) and shows for the first time that the association between empathy and sexual prejudice generalizes to the state level (P2), consistent with the emergent trait-level literature (Castiglione et al., 2013; Poteat et al., 2013; Burke et al., 2015; Marsden and Barnett, 2020). However, contrary to our expectations (P4), we found no evidence that participants displayed less state empathy toward gay/lesbian targets of the same gender as the participant compared to gay/lesbian targets of another gender (Kite and Whitley, 1996; Herek, 2000, 2002; Mahaffey et al., 2005; Herek and Gonzalez-Rivera, 2006; Ahrold and Meston, 2010; Herek and McLemore, 2013; Monto and Supinski, 2014). State empathy was negatively related to trait sexual prejudice, as predicted (P2) based on existing trait-level research (Bäckström and Björklund, 2007; Pettigrew and Tropp, 2008; Castiglione et al., 2013; Poteat et al., 2013; Sidanius et al., 2013; Burke et al., 2015; Marsden and Barnett, 2020; Stathi et al., 2021), and so was trait empathy (P5). However, this association was not unique to gay/lesbian conditions.

Our state empathy analyses may have been underpowered, as suggested by Bayes factors and observed power for the interactions. Furthermore, it is possible that the differences between straight and gay/lesbian conditions were too small for any interactions with target gender to be detected. Social identity theory states that prejudice occurs when members of a social outgroup are perceived as a threat to one’s ingroup identity (Turner and Reynolds, 2010). Research suggests that the degree to which LGBTQ+ people threaten heterosexual identity depends on the degree to which they conform to gender roles (Hamner, 1992; Basow and Johnson, 2000; Herek, 2002; Mahaffey et al., 2005; Glick et al., 2007; Vandello et al., 2008; Bosson et al., 2009; Ahrold and Meston, 2010; Gerhardstein and Anderson, 2010; Herek and McLemore, 2013; Monto and Supinski, 2014; Glotfelter and Anderson, 2017; Brassel and Anderson, 2020). Moreover, conformity to gender roles can modulate sexual prejudice responses (Monto and Supinski, 2014; Caswell and Sackett-Fox, 2018; Grigoryan et al., 2022). The vignettes used in the present study did not include characters that challenged traditional gender roles beyond having a same-sex partner, and may not have sufficiently threatened participants’ social identities to elicit large differences in empathy between straight and gay/lesbian conditions.

Relatedly, our state-level results may have been impacted by uncontrolled stereotype congruence through the randomized hobbies ascribed to each target. Although these were included simply to provide additional social context, some of these hobbies were stereotypically masculine (e.g., woodworking, hunting) while others were stereotypically feminine (e.g., knitting and baking) and others were not stereotypical of any gender (e.g., photography and swimming). The degree to which a gay/lesbian target violated traditional gender norms may have been modulated by their randomly assigned hobby. For instance, a gay man target may have elicited less social identity threat if their hobby was weightlifting compared to knitting. Since hobbies were not controlled for stereotypical masculinity/femininity across targets, this may have suppressed the expected interaction between target and participant gender through a lack of consistent gender norm violation from gay/lesbian targets. However, since hobby assignment was random we cannot definitively conclude that this contributed to our results without conducting a similar experiment in which stereotypical masculinity/femininity of hobbies is a controlled independent variable.

Our study is based on the framework used in studies of empathy and racial prejudice at the state level, which describe prejudice as being activated by certain diagnostic features of the target (e.g., skin color and facial features), leading to a corresponding down-regulation of empathic processing (Forgiarini et al., 2011; Kaseweter et al., 2012; Trawalter and Hoffman, 2015; Suleiman et al., 2018). Existing research on trait empathy and sexual prejudice reverses this view and conceptualizes empathy as a protective factor that inhibits prejudiced attitudes, leading to a reduction in prejudiced behaviors (Poteat et al., 2013; Burke et al., 2015; Marsden and Barnett, 2020). Although the present study is not a formal test of either model, our state-level results are more consistent with the former conceptualization. Testing the trait-level model of empathy and sexual prejudice at the state level poses significant practical challenges due to social desirability bias. Collecting state-level sexual prejudice ratings, for instance, may prime participants to report low levels of prejudice because they are explicitly asked whether they express attitudes that are socially undesirable. One option for circumventing this bias is implicit measures such as Implicit Association Tests, although these measures have been criticized in recent years (see Gawronski, 2019 for a review of the debate). Another option is to use measures of prejudiced behaviors, operationalized through choices in a simulated interaction or through coding naturalistic interactions. Future state-level research that seeks to directly test different conceptual models of empathy may require a shift in methods.

Even though we used simple, brief text vignettes, empathy ratings in the negative and neutral conditions were similar to those reported in the studies from which the vignettes were adapted, which coupled the vignettes with face images (McCrackin and Itier, 2021a, 2021b). Most studies of state empathy use faces expressing pain or other negative emotions (Derntl et al., 2010; Forgiarini et al., 2011; Montalan et al., 2012; Groen et al., 2013; Choi et al., 2014; Contreras-Huerta et al., 2014; Balconi and Canavesio, 2016; Fabi and Leuthold, 2016; Wang et al., 2016; Suleiman et al., 2018), or brief videos of painful or emotionally negative situations (Coll et al., 2012; Kaseweter et al., 2012; Meiring et al., 2014; Fourie et al., 2017; Martínez-Velázquez et al., 2020; Gehenne et al., 2021). When empathy-related vignettes are presented as text alone, the negative scenarios are typically long and elaborate (Arditte Hall et al., 2018; Hein et al., 2018; Vera Cruz and Mullet, 2019; McGrath and Haslam, 2020). Our results suggest that even simple vignettes can produce the expected main effect of empathy and a small but significant interaction between valence and target orientation.

However, our valence ratings were only slightly lower in negative conditions relative to neutral conditions, a result that deviates from previous studies (McCrackin and Itier, 2021a, 2021b). Without the added immersion of a visual stimulus to represent the characters or a greater degree of context, participants may not have been emotionally engaged by our stimuli. Participants may have engaged in cognitive empathy without having a strong affective sharing response to our vignettes. Such a distinction may reflect the broader differences between affective and cognitive empathy (Batson, 2009; Cuff et al., 2016). Future studies will need to carefully disentangle these constructs and better interpret participants’ state-level empathy.

Another potential explanation for our low valence ratings may lie in habituation to repeated emotional stimuli over the course of the experiment. Individuals who are repeatedly exposed to empathy-inducing stimuli and situations gradually deplete their ability to empathize, resulting in compassion fatigue (Hunt et al., 2017; Wilkinson et al., 2017; Coetzee and Laschinger, 2018; Cavanagh et al., 2020). To test whether this affected our results, we conducted a follow-up analysis as described in Supplementary material. We arranged participants’ state empathy ratings in temporal order and calculated the average rating in each quarter of the experiment, independent of condition. A repeated-measures ANOVA revealed no significant effect of time on state-level empathy ratings and a series of Pearson correlations demonstrated positive and significant correlations between empathy ratings in all quarters of the study. Therefore, we are confident that our state-level results are relatively stable across time and not substantially driven by compassion fatigue.

### 4.2 Associations between state empathy and trait empathy

Despite previous reports of associations between the TEQ and state measures of empathy such as mentalizing and social perception tasks (Spreng et al., 2009; Lima and Osório, 2021), no studies have yet reported TEQ association with self-reported empathy ratings obtained from a vignette design. Our results provide direct support to the notion that the TEQ is related to state measures of empathy (Spreng et al., 2009; Lima and Osório, 2021), using a novel state measure based on vignettes. Since the TEQ is believed to primarily reflect empathic concern (Spreng et al., 2009; Lima and Osório, 2021), this trait–state correlation suggests that individuals’ perception of their own empathy is strongly tied to their caring, concerned feelings for others. However, we cannot rule out the possibility that other constructs such as perspective-taking may also significantly inform self-perceptions of empathy, as the TEQ captures at least some variance related to this construct (Spreng et al., 2009; Lima and Osório, 2021). Further research is needed to parse out the contributions that different empathy constructs make toward state-level empathy.

### 4.3 Trait empathy and sexual prejudice

Most studies that demonstrate a negative association between sexual prejudice and trait empathy have used the IRI (Castiglione et al., 2013; Poteat et al., 2013; Burke et al., 2015; Marsden and Barnett, 2020). The present study is the first replication of this association using the TEQ, with a much smaller sample size than prior studies which ranged from 618 to 2044 participants (Poteat et al., 2013; Burke et al., 2015; Marsden and Barnett, 2020). This suggests that individuals who are highly prejudiced against LGBTQ+ people may be specifically lacking in empathic concern, which the TEQ mainly reflects (Spreng et al., 2009). Since sexual prejudice is fuelled by feelings of disgust (Shields and Harriman, 1984; Herek, 2000; Mahaffey et al., 2005; Olatunji, 2008; Inbar et al., 2009; Terrizzi et al., 2010; Herek and McLemore, 2013; Crawford et al., 2014; Kiebel et al., 2017; O’Handley et al., 2017), it seems fitting that individuals who score highly on measures of sexual prejudice are unlikely to be concerned for those they find disgusting. However, additional research is needed to determine whether it is sexual prejudice that fuels a deficit in empathy or vice versa, and whether disgust is at the core of this association.

Surprisingly, TEQ scores were significantly lower following the experiment relative to TEQ scores obtained during Mass Testing/prerequisite study and were not significantly correlated with each other. As TEQ2 was administered after completion of the experiment, participants’ ability to empathize may have been diminished. This would suggest that state-level influences can have an impact on supposedly trait-level measures. Indeed, it would suggest that a state-level empathy task would draw from the well of trait-level empathy, reducing the overall amount of trait empathy following the task. Of course, we cannot assume such causality from the present data and the same result could also simply be the product of low test–retest reliability. The studies reporting adequate test–retest reliability of the TEQ have used low sample sizes such as 65 or 77 participants (Spreng et al., 2009; Totan et al., 2012). Furthermore, it appears that scores on certain items of the TEQ are particularly variable across time (Xu et al., 2020). More stability analyses of the TEQ using very large samples are needed.

### 4.4 Limitations of the present work

The present study investigated only one facet of sexual prejudice and our results cannot directly speak to the association between empathy and prejudice against other groups who are often included under the umbrella of sexual prejudice, including other sexual minorities such as bisexual people, those who are marginalized due to their gender identity such as transgender and non-binary people, or individuals who face prejudice based on their association styles, such as non-monogamous people. Given that our samples were limited to young adults, our findings may not generalize to older adults; who are in particular more likely to exhibit high sexual prejudice (Herek and McLemore, 2013). Furthermore, we did not control for the personal memories and emotional associations our participants may have had to specific names, which may have biased their responses on some trials. Finally, our vignette and self-report empathy rating paradigm should not be assumed to generalize to real-world social situations or different state empathy behavioral tasks. Determining whether our findings replicate with different empathy tasks such as the Reading the Mind in the Eyes Test (Baron-Cohen et al., 2001), the Interpersonal Perception Task (Costanzo and Archer, 1989) and the Pictorial Empathy Test (Lindeman et al., 2018), is a potential avenue for future research.

### 4.5 Conclusion

Our state-level results suggest that the primary negative association between empathy and sexual prejudice persists at the state level, providing evidence beyond self-report trait scales. However, we did not observe modulation of this association by participant or target gender, suggesting our materials may not have produced a sufficiently large effect on which the influence of gender could be detected. Therefore, researchers interested in this topic should ensure that stimuli depicting LGBTQ+ people sufficiently challenge traditional gender roles and test the degree to which this influences results. Further research should also consider the different conceptual models used to describe the association between empathy and sexual prejudice and how these models can best be tested against one another at both the state and trait levels. We observed a promising novel correlation between state empathy and trait sexual prejudice, but further research is needed to determine which aspect of empathy drives this association. We also replicated and extended prior findings at the trait level using the TEQ, raising the question of which empathic constructs are most relevant in the context of sexual prejudice. The associations between empathy and sexual prejudice at both the trait and state level should be continued with diverse research designs, methodologies, and analytic tools to shed more light on the underlying mechanisms and develop strategies to address its real-world consequences.

## Data Availability

The raw data supporting the conclusions of this article will be made available by the authors, without undue reservation.

## Footnotes

^1^The text of the final item was “When I see someone being taken advantage of, I feel kind of protective toward him\her.”

^2^According to this interpretation, a *BF* between 1 and 3 indicates anecdotal evidence for the alternative hypothesis, a value between 3 and 10 indicates moderate evidence, a value between 10 and 30 indicates strong evidence, a value between 30 and 100 indicates very strong evidence, and any value greater than 100 indicates extreme evidence for the alternative hypothesis. Conversely, a *BF* between 1 and 0.333 indicates anecdotal evidence for the null hypothesis, a value between 0.333 and 0.100 indicates moderate evidence, a value between 0.100 and 0.033 indicates strong evidence, a value between 0.033 and 0.010 indicates very strong evidence, and a value below 0.010 indicates extremely strong evidence for the null hypothesis.

^3^The interaction between target gender, participant gender, and group was the only significant interaction, *F*(1, 197) = 5.652, *MSE* = 2.084, *p* = 0.018, *η*^2^ = 0.028. However, based on simple effects testing, this small interaction was driven by female MTurk participants (*M* = 6.415, *SD* = 0.133) rating their empathy slightly higher than female SONA participants (*M* = 6.029, *SD* = 0.153) during vignettes with a female target, *F*(1, 197) = 4.094, *MSE* = 3.921, *p* = 0.049, *η*^2^ = 0.020, which did not compromise our grouping of the two samples.

^4^A supplementary ANOVA with the same factors performed on valence ratings also found a main effect of valence, *F*(1, 199) = 11.128, *MSE* = 143.165, *p* = 0.001, *η2 =* 0.053. This effect was driven by slightly more negative valence in the negative conditions (*M* = 4.304, *SE* = 0.148) than the neutral conditions (*M* = 4.901, *SE = 0*.075), as expected. No other effects or interactions were significant.
