# Supplementary material for: The influence of sexual prejudice and gender on trait and state-level empathy
Source: Front Psychol. 2025 Apr 1;16:1527124. doi: 10.3389/fpsyg.2025.1527124 (PMC11996783; doi:10.3389/fpsyg.2025.1527124)
Supplement: Supplementary file 1 [file Data_Sheet_1.pdf]

Supplementary Material

1 Supplementary Analysis

The following analysis was conducted to determine whether participants' responses were influenced by habituation to the repetitive, emotionally negative content of our vignettes. There is an extensive literature on compassion fatigue, a phenomenon by which individuals' ability to empathize with others is depleted over time and repeated exposure to distress and suffering (Hunt et al., 2017; Wilkinson et al., 2017; Coetzee and Laschinger, 2018; Cavanagh et al., 2020). We wanted to ensure that our state-level results were not confounded by this effect and that our state empathy ratings were temporally stable over the course of the experiment, especially given the generally weak differences in valence ratings between negative and neutral conditions.

To this end, we arranged each participants' empathy ratings on each trial by temporal order and calculated their average empathy ratings in the first, second, third, and fourth quarters of the experiment. We then conducted a repeated-measures ANOVA with one factor (Time) and four levels (Quarter 1, Quarter 2, Quarter 3, Quarter 4) to determine whether empathy ratings changed over time. Note that the assumption of sphericity was violated (Mauchly's $W = 0.550$, $p < .001$) so the following results are reported with the Greenhouse-Geisser correction applied. As seen in Supplementary Figure 1., we found no significant effect of time, $F(2.23{,}445.23) = 0.80$, $MSE = 0.35$, $\eta^2 < .01$, $BF = 0.01$, $Power = .64$.

2 Supplementary Figures

Supplementary Figure 1

Non-significant effect of time on mean state empathy ratings, across all conditions. $N = 201$.

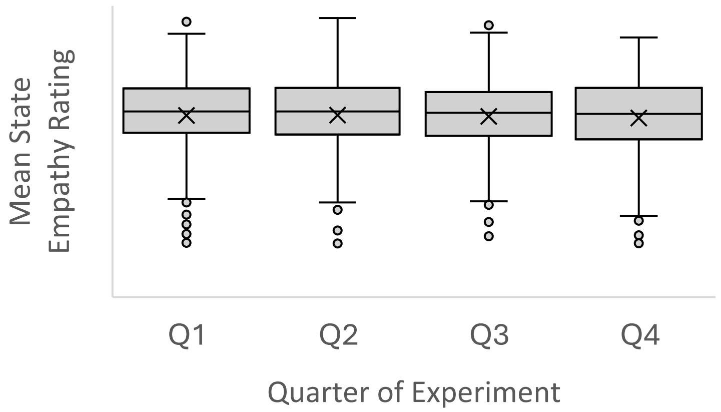

3 Supplementary Tables

Next, we conducted a series of Pearson correlations to determine how average state empathy ratings in each quarter of the experiment were associated with each other, irrespective of their raw values. These correlations revealed that average ratings in all four quarters were positively and significantly associated with one another (Supplementary Table 1).

Supplementary Table 1

Significant Pearson correlations and Bayes factors for associations between mean state empathy ratings in each quarter of the experiment. All p-values < .001, observed power for all tests was 1.00. $N = 201$.

Quarter	Statistic	Q1	Q2	Q3
Q1	*r*	-	-	-
	BF	-	-	-
Q2	*r*	.80	-	-
	BF	1.13×10^{42}	-	-
Q3	*r*	.69	.89	-
	BF	6.51×10^{26}	1.90×10^{66}	-
Q4	*r*	.59	.75	.80
	BF	2.76×10^{17}	2.70×10^{34}	4.01×10^{43}

The results of these supplementary analyses suggest that our participants were not meaningfully influenced by compassion fatigue over the course of the experiment and that our state empathy ratings were generally stable across time.
